# The role of Hashimoto thyroiditis in predicting radioiodine ablation efficacy and prognosis of low to intermediate risk differentiated thyroid cancer

**DOI:** 10.1007/s12149-021-01644-1

**Published:** 2021-06-21

**Authors:** Domenico Albano, Francesco Dondi, Valentina Zilioli, Maria Beatrice Panarotto, Alessandro Galani, Carlo Cappelli, Francesco Bertagna, Raffaele Giubbini, Claudio Casella

**Affiliations:** 1 Nuclear Medicine, University of Brescia and ASST Spedali Civili Brescia, Brescia, Italy; 2 Nuclear Medicine Department, ASST Spedali Civili Brescia, Brescia, Italy; 3 Department of Clinical and Experimental Sciences, Surgical Clinic, University of Brescia, Brescia, Italy; 4 Department of Clinical and Experimental Sciences, SSd Medicina ad Indirizzo Endocrino-Metabolico, University of Brescia, Brescia, Italy; 5 Department of Molecular and Translation Medicine, Surgical Clinic, University of Brescia, Brescia, Italy

**Keywords:** RAI, Differentiated thyroid cancer, Hashimoto thyroiditis, Ablation

## Abstract

**Objective:**

The baseline treatment of differentiated thyroid cancer (DTC) consists of thyroidectomy followed by postoperative risk-adapted radioiodine therapy (RAIT) when indicated. The choice of most appropriate RAI activities to administer with the aim to reach an efficient remnant ablation and reduce the risk of recurrence is yet an open issue and the detection of basal factors that may predict treatment response seems fundamental. The aim of this study was to investigate the potential role of Hashimoto thyroiditis (HT) in predicting 1-year and 5-year treatment response after RAIT and prognosis.

**Methods:**

We retrospectively included 314 consecutive patients (174 low-risk and 140 intermediate-risk) who received thyroidectomy plus RAIT. One-year and 5-year disease status was evaluated according to 2015 ATA categories response based upon biochemical and structural findings.

**Results:**

HT was reported histopathologically in 120 patients (38%). DTC patients with concomitant HT received a higher number of RAITs and cumulative RAI activities. Initial RAIT reached an excellent response in 63% after one year and 84% after 5 years. The rate of excellent response one year and 5-year after first RAIT was significantly lower in HT groups, compared to not HT (*p* < 0.001). Instead, HT did not have a prognostic role considering PFS and OS; while stimulate thyroglobulin (sTg) at ablation was significantly related to survival.

**Conclusions:**

HT may affect the efficacy of RAIT in low to intermediate risk DTC, particularly reducing the successful rate of excellent response after RAIT. Instead, HT did not have a prognostic impact such as stimulated sTg.

## Introduction

Differentiated thyroid cancer (DTC) is the most common endocrine malignancy, accounting for 1% of all cancers diagnosed each year [1, 2] and it is considered a slowly growing cancer with an overall good outcome, except of cases with distant metastases or radioiodine-refractory disease [3, 4]. The baseline treatment of DTC consists of thyroidectomy followed by postoperative risk-adapted radioiodine therapy (RAIT) when indicated [2, 5].

The choice of most appropriate RAI activities to administer with the aim to reach an efficient remnant ablation and reduce the risk of recurrence is yet an open issue. This point is even more debated in low-risk and intermediate-risk DTC, where it seems crucial to find the best balance between the effective cure and the risk of side effects (reduced radiation exposure, cost, time of hospitalization) [6–9]. Consensus guidelines recommend a dose of 1.1–3.7 GBq for patients with low to intermediate-risk DTC and higher activities for high-risk DTC, residual disease or aggressive histological subtypes [2]. Several papers [10, 11] demonstrated the equivalence of successful ablation between low-activity (1.1 GBq) and high-activity (3.7 Gbq) and between thyroid hormone withdrawal and recombinant human thyroid-stimulating hormone injections, without significant difference in outcome survival.

To better understand which activity inject in these patients successfully while minimizing the side effects, it seems fundamental to know the factors that influence the success of ablation and to predict the outcome.

The most shared known factors include the preablation stimulated thyroglobulin (sTg) level, the completeness of surgery, the preablation radioiodine scan findings and the histotype [12–14]. The potential influence of Hashimoto Thyroiditis (HT) in the effectiveness of RAIT is not well-investigated. Only Kwon et al. [15] showed that coexisting HT together with sTg were negative predictive factors for successful low-dose RAIT (1.1 GBq).

Moreover, also in predicting treatment response and prognosis the role of HT is not well-investigated. The association between DTC and HT has been long suggested with several studies in favor [16–18]. Probably, HT is frequently associate with DTC, especially papillary thyroid carcinoma, and may be considered a risk factor for developing thyroid cancer but the reason of this link is not yet understood [19, 20]. Moreover, DTC patients with concomitant HT seems to have a better prognosis that DTC patients without [21, 22].

The main aim of this study was to retrospectively investigate in a consecutive cohort of patients with low- to intermediate-risk DTC treated with total thyroidectomy and RAIT, the role of HT in predicting 1-year and 5-year treatment response and disease status. The second aim was to analyze the potential prognostic role of HT in this population.

## Materials and methods

We retrospectively screened all patients who performed thyroidectomy from January 2010 until January 2018 in our Surgery Department. Inclusion criteria were (1) a total thyroidectomy followed by RAIT, (2) age more or equal to 18 years at time of surgery, (3) a histological confirmation of DTC, (4) a classification as low risk or intermediate risk according to 2015 ATA guidelines [2], (5) at least 12 months of clinical and imaging follow-up after RAIT. Exclusion criteria were (1) age less than 18-years old, (2) hypothyroidism or hyperthyroidism condition before surgery, (3) a previous history of other neoplastic disease. Finally, 314 patients were included: 174 low-risk class stage after surgery and RAIT and 140 intermediate-risk class. There was a prevalence of female (F:M = 2.8:1) and mean age at diagnosis was 50-years old (Table 1). The first administered activity of RAI ablation ranged from 1.1 to 4.4 GBq (average 2.7 GBq) and it was established according to the risk class based on the TNM staging of the AJCC currently in use. Patients treated before the introduction of new American Joint Committee on Cancer/International Union against Cancer (AJCC) 8th edition were reclassified according to the last guideline [23]; this explains why some patients were treated with high-activities due to the downstage from intermediate-risk class to low-risk one.

**Table 1:** Baseline features of our population

	Average ± SD (range)	Patients *n* (%)
Age years	50 ± 14 (18–85)	
Gender		
Male		82 (26%)
Female		232 (74%)
Histotype		
Papillary		146 (46%)
Follicular variant of papillary		105 (34%)
Follicular		25 (8%)
Aggressive variant (tall cells and sclerosing diffuse)		26 (8%)
Hurtle cell		12 (4%)
Lymphatic invasion		91 (29%)
Vascular invasion		76 (24%)
Extrathyroidal extension		110 (35%)
Resection margin involvement		49 (16%)
Tumor size (mm)	18.4 ± 15.2 (1–85)	
Multicentricity		160 (51%)
Hashimoto thyroiditis		120 (38%)
T-stage		
sT1		179 (62%)
sT2		47 (10%)
sT3		88 (28%)
N-stage		
sN0		240 (78%)
sN1a		30 (9%)
sN1b		44 (14%)
ATA class risk		
Low		174 (55%)
Intermediate		140 (45%)
Pre-ablation Tg	1.8 ± 2.2 (0.1–100)	
sTg at the time of ablation (ng/mL)	10.5 ± 25.9 (0.1–250)	
TgAb positive at ablation		114 (36%)
First RAI activities administrated (GBq)	2.7 ± 1.2 (1–4.4)	
Cumulative RAI activities administrated (GBq)	6.3 ± 8 (1–56)	
No. therapies	1.5 ± 1 (1–7)	
1-year treatment-response categories		
Excellent response		199 (63%)
Indeterminate response		53 (17%)
Biochemical and/or structural incomplete response		62 (20%)
5-year treatment–response categories		
Excellent response		264 (84%)
Indeterminate response		12 (4%)
Biochemical and/or structural incomplete response		19 (6%)
Progression		4 (1%)
Na		15 (5%)

*n* number, *GBq* Gigabequerel, *RAI* radioiodine, *var* variant, *sTg* stimulated thyroglobulin, *Ab* antibodies, *na* not available

All patients underwent total thyroidectomy and had a histopathological diagnosis of DTC: 146 classic variant of papillary carcinoma, 105 follicular variant of papillary carcinoma, 25 follicular carcinoma (17 minimally invasive and 8 widely invasive), 26 aggressive papillary variants (18 tall cells variant of papillary carcinoma and 8 sclerosing diffuse variant of papillary carcinoma) and 12 Hürthle cell carcinoma. One hundred and fifty patients underwent also a central lymphadenectomy and also lateral in fifty-four cases. HT was reported at the histological sample in 120 patients (38%), while in the remaining 194 cases (62%) no foci of HT were described. All patients received RAIT 2–3 months after thyroidectomy: 137 patients discontinued levothyroxine treatment 4 weeks before radioactive-iodine remnant ablation and replaced by triiodothyronine for 2 weeks, while 177 patients received recombinant human thyrotropin (rhTSH-Thyrogen, Genzyme Corporation) intramuscularly with a dose of 0.9 mg over 2 consecutive days during treatment with levothyroxine and RAI was administered the day after the second injection. Serum thyrotropin (TSH), fT3, fT4, Tg, antithyroglobulin antibodies (TgAb), and ioduria were measured before RAIT. All patients followed a low-iodine diet for at least 2 weeks and mean TSH level was higher than 30 UI/L in all cases. Three days after RAIT, the patients underwent a whole body scintigraphy (WBS) followed by single photon emission computed tomography/computed tomography (SPECT/CT), by hybrid dual-detector SPECT/CT (Infinia Hawkeye II, GE Healthcare, Haifa Israel), equipped with 1 inch StarBrite^TM^Crystal and a high-energy collimator. The StarBrite™ Crystal has a sensitivity more than twofolds higher than that provided by a 3/8 inch crystal without a significant loss in resolution, therefore contributing to improve the reliability of SPECT with RAI. The WBS was performed in continuous mode with high-energy general purpose (HEGP) parallel holes collimator, 364-keV photopeak with ± 10% energy windows setting and scatter correction. The infrared-based real-time automatic body contouring system was activated for simultaneous anterior and posterior view with a matrix of 256 × 1024. Hybrid SPECT/CT scans from skull base to the lung bases were routinely acquired in all cases and additional SPECT/CT scans of other areas were done according to the WBS findings. SPECT images were acquired with HEGP collimator, matrix size of 128 × 128, 364-keV photopeak with ± 10% energy and scatter windows, dual-detector 180° acquisition, angular step of 3°, 15″ time per step/view. The CT parameters were 140 kV, 2.5 mA, 30″ rotation speed, 10 mm slice thickness, 256 × 256 matrix. CT acquisition was performed with a 2-slices elicoidal acquisition. An ordered subset expectation maximization iterative reconstruction with CT-based attenuation correction and scatter correction was performed. Moreover, about one-year after RAIT, a WBS after a diagnostic activity of 185 MBq or after a therapeutic activity in case of persistent disease was executed. Also sTg, TSH and TgAb were done at the same time and the combination of biochemical and radiological findings were used to classify the ablation success rate and the treatment response categories according to ATA groups (excellent response, biochemical incomplete response, structural response and indeterminate response) [2]. Disease status 5 year after RAIT was evaluated considering a combination of clinical, biochemical (Tg, AbTg) and imaging (ultrasound, CT, WBS and/or PET/CT) data.

### Statistical analysis

All statistical analyses were carried out using Statistical Package for Social Science (SPSS) version 23.0 for Windows (IBM, Chicago, Illinois, USA) and MedCalc Software version 17.1 for Windows (Ostend, Belgium). The descriptive analysis of categorical variables comprised the calculation of simple and relative frequencies; the numeric variables were described as mean, standard deviation, minimum and maximum.

The statistical significance of the categorical variables was tested with *χ*^2^ or Fisher’s exact test and a Student’s *t*-test or Mann–Whitney’s *U*-test were performed for the continuous features. A *p* value < 0.05 was considered statistically significant.

Progression-free survival (PFS) was calculated from the date of diagnosis to the date of first disease progression, relapse, death or the date of last follow-up. Overall survival (OS) was calculated from the date of diagnosis to the date of death from any cause or to the date of last follow-up. Survival curves were plotted according to the Kaplan–Meier method and differences between groups were analyzed using a two-tailed log rank test. Cox regression was used to estimate the hazard ratio (HR) and its confidence interval (CI). A *p* value < 0.05 was considered statistically significant.

## Results

### Treatment response evaluation

Patients were divided in two groups according to the presence of HT: group 1 consists of 194 (62%) patients without HT and group 2 of 120 (38%) with HT concomitant to DTC (Table 2).

**Table 2:** comparison between DTC patients with concomitant HT and without

Variable	no HT *n* 194	HT *n* 120	*p* value
Age, mean (years)	51.3	48.2	0.058
Gender F:M	139:55	93:27	0.252
Histotype			0.090
Papillary	86 (44%)	60 (50%)	
Follicular variant of papillary	65 (33%)	40 (33%)	
Follicular	22 (11%)	3 (3%)	
Aggressive variant	13 (7%)	13 (11%)	
Hurtle cell	8 (4%)	4 (3%)	
Lymphatic invasion	54 (28%)	37 (31%)	0.570
Vascular invasion	46 (24%)	30 (25%)	0.796
Extrathyroidal extension	67 (35%)	43 (36%)	0.891
Resection margin involvement	33 (17%)	16 (13%)	0.385
Tumor size (mm)	18.3	18.8	0.747
Multicentricity	95 (49%)	65 (54%)	0.372
ATA class risk			0.295
Low	112 (58%)	62 (51%)	
Intermediate	82 (42%)	58 (49%)	
Lymph node metastasis	41 (21%)	34 (28%)	0.289
Pre-ablation Tg (ng/mL)	1.9	1.8	0.887
sTg at the time of ablation (ng/mL)	8.4	7.5	0.470
TgAb positive at ablation	46 (24%)	68 (57%)	< 0.001
First RAI activities administrated (GBq)	2.6	2.8	0.242
Cumulative RAI activities administrated (GBq)	5.1	8.2	< 0.001
N° therapies	1.3	1.7	0.001
1-year treatment–response categories			< 0.001
Excellent response	134 (69%)	65 (54%)	
Not Excellent response	60 (31%)	55 (46%)	
5-year treatment–response categories			< 0.001
Excellent response	171 (94%)	93 (79%)	
Not Excellent response	11 (6%)	24 (21%)	
Data non available	12	3	
Recurrence	11 (6%)	12 (10%)	0.153
Death	3 (2%)	2 (2%)	0.934

Considering the main epidemiological (age, gender), histological (histotype, lymphatic invasion, vascular invasion, extrathyroidal invasion, resection margin involvement, multicentricity and tumor size) and clinical features (ATA class risk, nodal disease at staging, pre-ablation Tg and sTg) not significant differences were present between the two groups. On the other hand, the presence of AbTg was significantly higher in HT groups than non HT (57% vs 24%, *p* < 0.001).

Also cumulative RAI activities administrated and number of radiometabolic therapies were higher in patients with coexisting HT than those without HT (*p* < 0.001 for both).

**Fig. 1:**
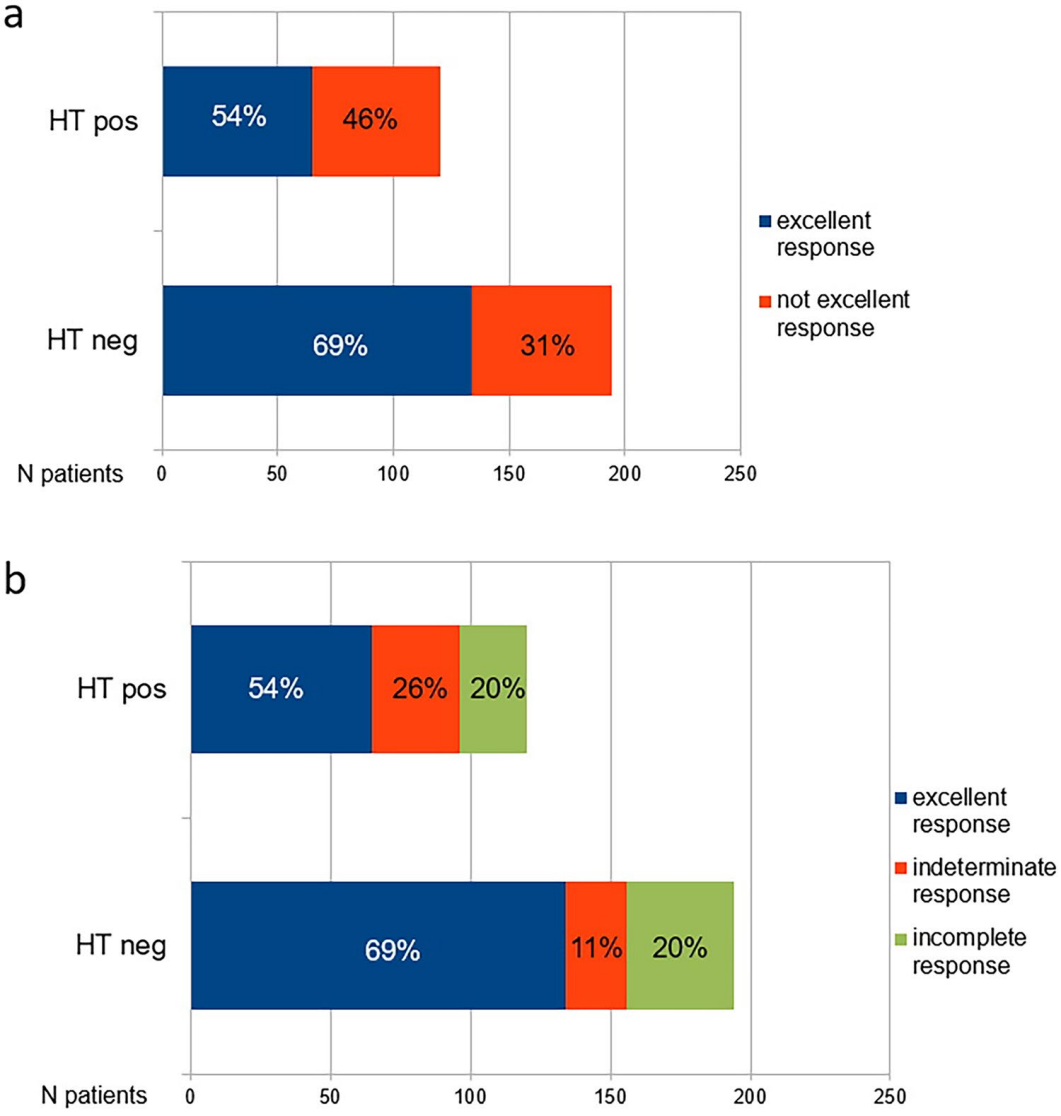
Histogram distribution of treatment response categories one year after first RAIT

Most of patients (63%) had an excellent response one year after RAIT, 17% an indeterminate response, 20% an incomplete response (5% only biochemical; 2% only structural and 13% both biochemical and structural) (Table 1). Patients with coexisting HT showed a lower rate of 1-year excellent response (54%) than without HT (69%) (*p* < 0.001) (Fig. 1a). In patients without HT, 1-year not excellent response consisted of 21 cases of indeterminate response and 39 of incomplete response (27 both biochemical and structural; 7 only biochemical and 5 only structural). While, in patients with HT, 1-year not excellent response consisted of 31 cases of indeterminate response and 24 of incomplete response (20 both biochemical and structural; 3 only biochemical and 1 only structural) (Fig. 1b).

Comparing neck ultrasound findings 6–12 months after first radiometabolic therapy, no differences were described between the two groups (HT vs no HT).

Considering disease status five year after RAI first therapy, the rate of excellent response was 84%, of indeterminate response 4%, incomplete response 6% and progression 1%. In 15 patients the 5-year disease status was not available.

**Fig. 2:**
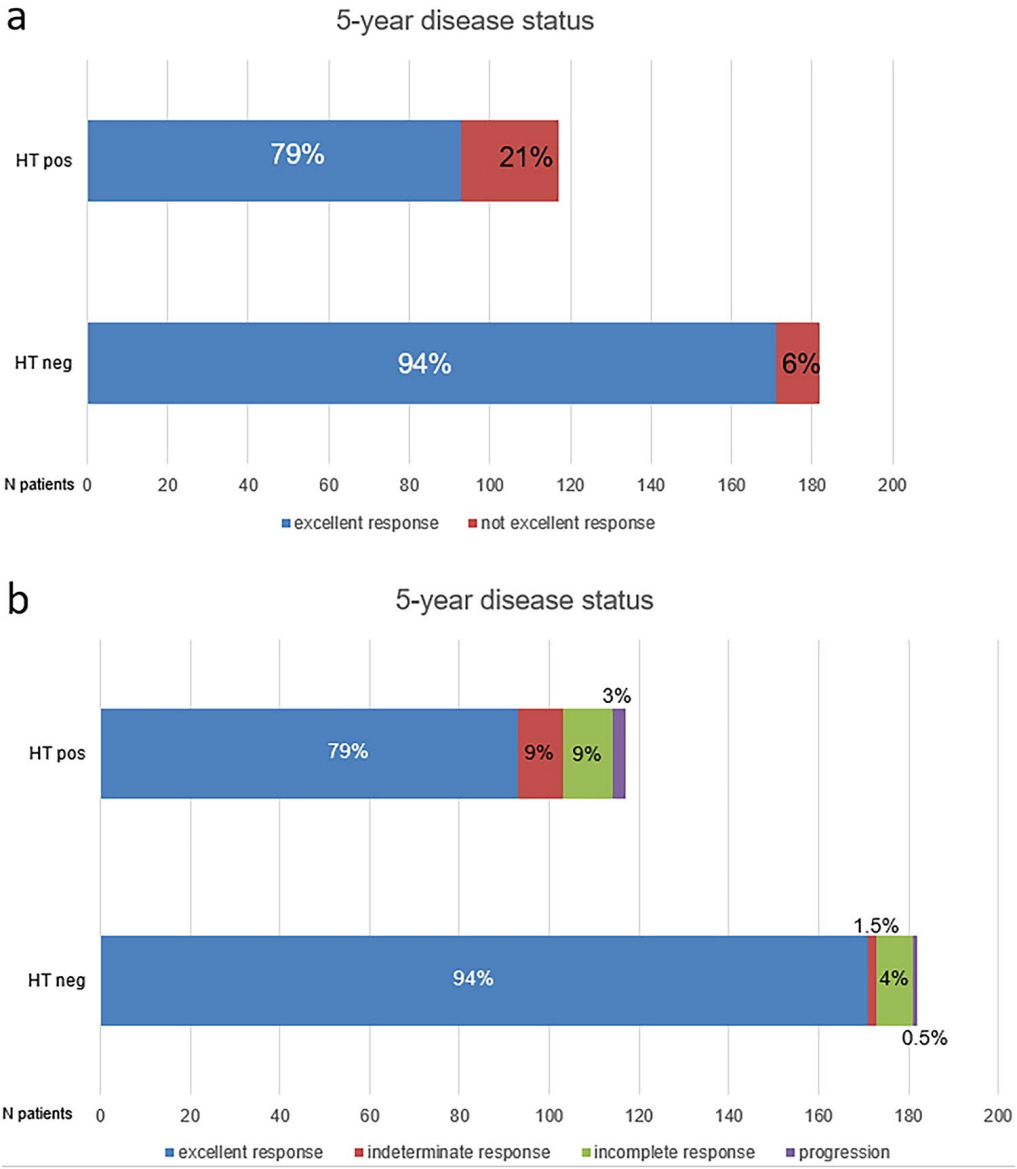
Histogram distribution of treatment response categories 5 years after first RAIT

Patients with coexisting HT showed a lower rate of 5-year excellent response (79%) than without HT (94%) (*p* < 0.001) (Fig. 2a). In patients without HT, 5-year not excellent response consisted of 3 cases of indeterminate response, 7 of incomplete response (6 both biochemical and structural and 1only biochemical) and one of progression. Instead in patients with HT, 5-year not excellent response consisted of 10 cases of indeterminate response, 10 of incomplete response (7 both biochemical and structural; 2 only biochemical and 1 only structural) and 4 of progression (Fig. 2b).

### Prognosis

**Fig. 3:**
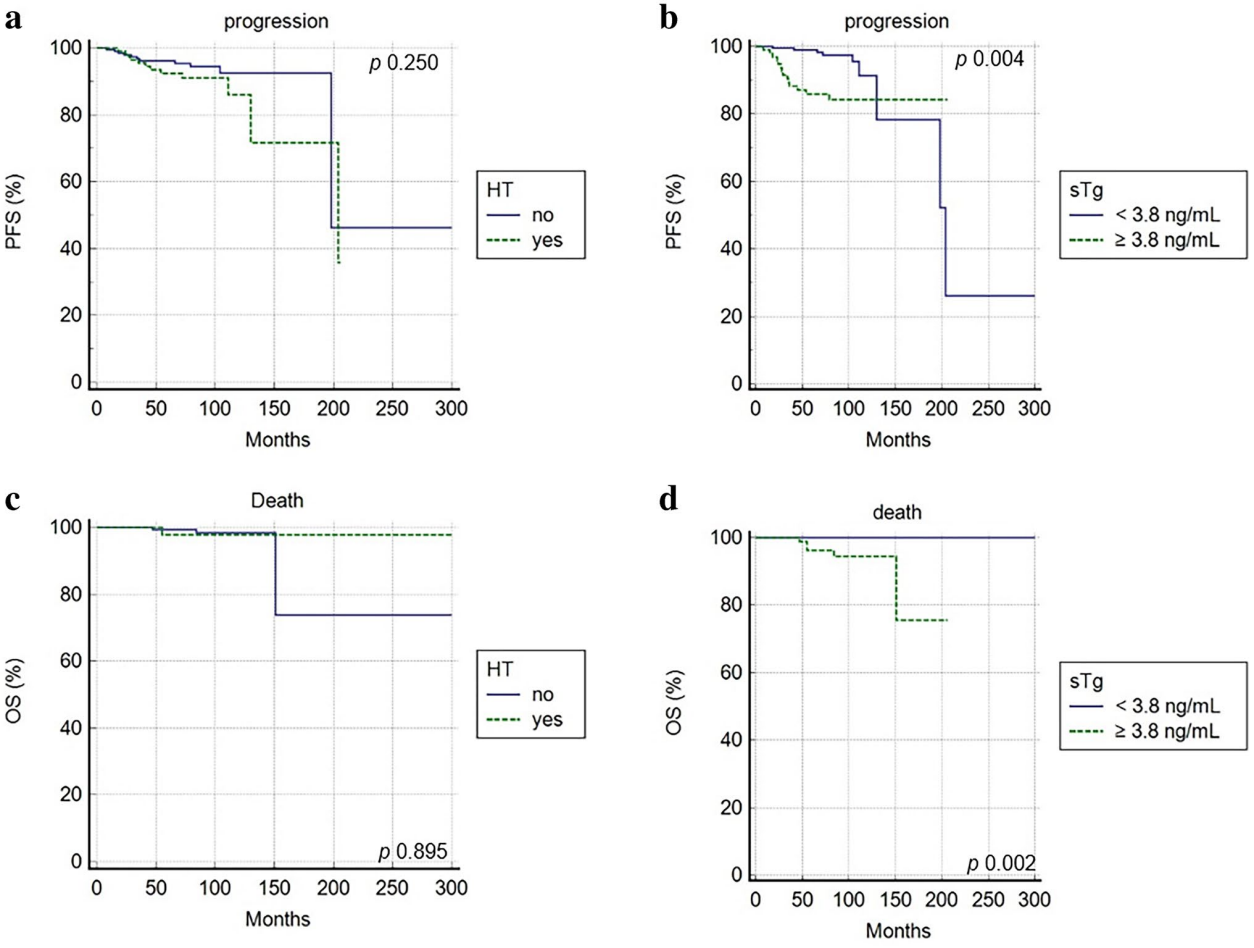
Progression free survival **a, b** and overall survival **c**, **d** according to Hashimoto thyroiditis (HT) and stimulated thyroglobulin (sTg)

At a median follow-up of 88 months, relapse or progression of disease occurred in 23 patients with an average time of 60.8 months (8–204 months), while death occurred in 5 patients with an average time of 78.4 months (55–151). The median PFS was 85 months (8–300 months) and the median OS was 88 months (10–300 months). In univariate analysis, gender, age, ATA class risk group, sTg and disease status after 1 and 5 years are significantly related with PFS (Table 3). Stimulated Tg was dichotomized using ROC curve analysis and 3.8 ng/mL was derived as best threshold (AUC 0.775, sensitivity 100%, specificity 71%, *p* < 0.001). The other clinical and pathological features, like HT, were not significantly associated with PFS. In multivariate analysis, age, sTg, 1-year disease status and 5-year disease status were confirmed to be independent prognostic factors for PFS (Fig. 3).

**Table 3:** Univariate and multivariate analyses for PFS and OS

	Univariate analysis		Multivariate analysis	
	*p* value	HR (95% CI)	*p* value	HR (95% CI)
PFS				
Gender	0.046	0.398 (0.160–0.987)	0.251	2.222 (0.859–4.520)
Age (< 55 years vs ≥ 55)	0.004	0.282 (0.117–0.683)	0.004	3.590 (1.482–8.691)
ATA class risk (low vs intermediate)	0.023	0.274 (0.119–0.629)	0.281	2.945 (0.345–7.728)
Histotype	0.210	2.123 (0.872–5.449)		
HT	0.250	0.610 (0.269–1.416)		
Multifocality	0.584	0.772 (0.332–1.794)		
Vascular invasion	0.600	0.998 (0.235–2.987)		
Lymphatic invasion	0.251	0.567 (0.215–1.495)		
Tumor size	0.187	0.443 (0.100–1.435)		
TgAb presence	0.298	1.580 (0.666–3.748)		
sTg at ablation (cutoff 3.8 ng/mL)	0.004	0.278 (0.116–0.668)	0.027	2.748 (1.151–6.560)
1-year disease status*	0.003	0.201 (0.085–0.475)	0.009	1.540 (1.222–1.879)
5 year disease status*	0.001	0.076 (0.021–0.273)	0.010	2.435 (1.200–5.987)
OS				
Gender	0.304	0.252 (0.041–1.531)		
Age	0.040	0.148(0.002–0.922)	0.198	1.876 (0.546–3.454)
ATA class risk	0.134	0.252 (0.041–1.531)		
Histotype	0.657	1.694 (0.235–12.398)		
HT	0.895	1.127 (0.183–6.826)		
Multifocality	0.879	1.151 (0.186–7.125)		
Vascular invasion	0.090	0.183 (0.0241.335)		
Lymphatic invasion	0.078	0.163 (0.021–1.227)		
Tumor size	0.224	0.198 (0.098–1.987)		
TgAb presence	0.070	na		
sTg at ablation	0.002	na	0.034	1.890 (1.121–2.876)
1-year disease status*	0.345	0.415 (0.065–2.596)		
5 year disease status*	0.545	0.418 (0.025–6.937)		
Total no. RAITs	0.039	na	0.212	1.333 (0.879–2.001)
Cumulative RAI activities administrated (GBq)	0.011	na	0.234	1.010 (0.768–2.221)

*PFS* progression-free survival, *OS* overall survival, *HR* hazard ratio, *CI* confidence interval

*Variables dichotomized as excellent response vs not excellent response

While considering OS, at univariate analysis age, sTg, cumulative RAI activities administered and total number of RAIT were significantly correlated with death. At multivariate analysis, only sTg was confirmed to be an independent prognostic variable (Table 3; Fig. 3).

## Discussion

HT is the most common autoimmune inflammatory thyroid disease with a worldwide annual incidence of 0.3–1.5 cases for 1000 persons and it is considered the first cause of hypothyroidism in the iodine-sufficient areas of the world [24]. The etiopathogenesis is probably a combination between environmental factors and genetic condition which causes the loss of immunological tolerance, with a subsequent autoimmune attack to the thyroid tissue. HT brings a chronic inflammatory condition that awakens an immune response leading to a continuous damage of surrounding stromal cells; overall this phenomenon causes potential genetic alterations, an inappropriate cell proliferation, and increases the risk of neoplastic transformation. Historically, HT was demonstrated significantly associate with an increased risk of developing thyroid lymphoma [25, 26]. But also the association between HT and DTC has been investigated with positive evidences [19, 20]. First in 1955 Dailey et al. [27] reported an increased association between HT and DTC, mainly papillary TC. However, the reason of this link remains yet unclear. The incidence of HT with concomitant PTC were reported as 26.8–38.1% in Italy, and 14.7–30.1% in the United States [28]. There are present in literature several hypotheses: someone suggested that HT could be a “cancer-impending effect” meaning an attempt of autoimmune system to fight against the tumor with the aim to cure the oncological disease and it could explain the favorable outcome reported in DTC with concomitant HT [29]; others speculated that the elevated TSH levels, often secondary to HT, might stimulate follicular epithelial cells proliferation and gained also the proliferation of cancer cells [30], but no shared consensus is available.

In our sample, the two groups (HT vs. no HT) had similar epidemiological and histological features, despite the histotype variants distribution and the age were near to statistical significance (0.090 for histotype and 0.058 for age). Patients with HT had lower age at diagnosis and less cases of follicular DTC in comparison with no HT patients.

Beside the linkage between HT and DTC, it seems crucial to clarify if conventional therapy of DTC may be affected by the presence or not of HT. Kwon et al. [15] conducted a retrospective analysis on 691 DTC patients who underwent thyroidectomy plus 1.1 GBq of RAI. Successful RAI ablation was registered in 62.3% of patients, and stimulated Tg at ablation and coexisting HT were the only two factors significantly associated with the success rate. The efficacy of low-activity of RAI was negatively influenced by sTg and HT. Moreover, patients with HT underwent more RAI therapies and higher total RAI activity. These evidences were confirmed also by our analysis: in DTC patients with coexisting HT the rate of 1-year excellent response was lower than DTC patients without HT (54% vs 69%; *p* < 0.001) and also the 5-year excellent response was lower than DTC patients without HT (79% vs 94%; *p* < 0.001). One possible explanation for RAI failure is the reduction of lower sodium–iodide symporter (NIS) expression in patients with coexisting HT, which it is strictly related to the decreased radioactive iodine uptake into the remnant thyroid tissue [31]. Kollecher et al. [32] demonstrated that thyroid tissue with HT showed significantly lower levels of NIS membrane staining than normal thyroid tissue. Moreover, the inflammatory background typical of HT, may reduce the therapeutic efficacy of RAI. Also for high-activity (3.7 GBq) HT seems to have a significant role in predicting ablation success after RAI [33]: in patients with HT the success rate was 33.4% in comparison with DTC without HT with a rate of 66.6%. In our study, we included both low and intermediate risk class DTC, thus low–intermediate RAI activity was injected. The presence of HT was related with higher cumulative RAI activities administrated and higher number of radiometabolic therapies; also in this case, the reason could be related with the less efficacy of radioiodine to enter in the thyroid cells due to the inflammatory micro-environment and the loss NIS expression.

In the last 2015 ATA guidelines [2], for the first time a new category response was introduce called indeterminate response which consist of patients without clear biochemical or structural disease, but without a complete response. In this group it is included also the case of DTC patient with TgAb stable or declining, but positive, without definitive structural evidence of disease. Two studies have demonstrated that only 13–20% of patients with an indeterminate response to therapy are reclassified as persistent/recurrent disease over approximately 10 years of follow-up [34, 35]

The rate of patients with indeterminate response after RAIT was significantly higher in HT patients compared to not HT both 1 year and 5 years after RAIT. This is directly associate with the presence of TgAb in patients with HT compared in patients without. The high rate of indeterminate response could partially explain the different excellent response rate between the two groups, even though the prognosis is not affected.

On the other hand, HT does not show a prognostic role in predicting PFS and OS. Other parameters, like age at diagnosis, sTg, and disease status after first-line therapy were significantly correlated with the risk of recurrence. Stimulated Tg confirmed to be an independent prognostic variable also for OS. Several papers underlined a possible association between HT and a good outcome, describing that DTC with concomitant HT had a lower rate of lymph node and distant metastases [22, 36, 37]. In our analysis, no significant impact of HT in prognosis was showed also due to the selection of low- to intermediate-risk DTC, which for definition have a good prognosis. In fact, the number of events (progression/relapse or death) was relatively low. As previously demonstrated [2], preablation sTg level has been considered a poor prognostic factor for successful ablation and prognosis. In this manuscript, a sTg threshold of 3.8 ng/mL was derived as the best compromise between sensitivity and specificity; other cutoff values are suggested in literature [38, 39].

Our study had several limitations, including the retrospective nature of the study design, the heterogeneity of patients’ features and the relatively not long follow-up time (88 months in this study) for prognostic evaluation considering the outcome of low to intermediate risk DTC.

In conclusion, we have demonstrated that HT may affect the efficacy of RAIT in low- to intermediate-risk DTC, particularly reducing the successful rate of excellent response after RAIT. Instead, HT does not seem to have a prognostic role considering PFS and OS.
